# Relational Wellness in Brief Medical and Dental Feedback: The Proposed SEED Dialogue Heuristic

**DOI:** 10.3390/healthcare14213559

**Published:** 2026-10-22

**Authors:** Roopwant Kaur, Rosa Chaviano-Moran, Salwa Mekled, Salma Elwazeer

**Affiliations:** 1 Department of General Dentistry, School of Dental Medicine, East Carolina University, Greenville, NC 27834, USA; 2 Rutgers School of Dental Medicine, Rutgers Biomedical and Health Sciences, Newark, NJ 07103, USA; chaviaro@sdm.rutgers.edu; 3 Restorative Dentistry, Maurice H. Kornberg School of Dentistry, Temple University, Philadelphia, PA 19140, USA; salwa.mekled@temple.edu; 4 Department of Dental Public Health, Woody L. Hunt School of Dental Medicine, Texas Tech University Health Sciences Center, El Paso, TX 79905, USA; salma.elwazeer@ttep.edu

**Keywords:** clinical feedback, dental education, feedback dialogue, feedback literacy, health professions education, medical education, psychological safety, relational wellness, SEED

## Abstract

**Highlights:**

**What are the main findings?**

- Relational wellness is proposed as a dyadic condition of a particular feedback episode: both participants can remain respected and connected while addressing performance truthfully; its dimensions and distinctness require testing.
- The proposed SEED dialogue heuristic is a four-move, author-developed prototype for opening brief feedback conversations; it is not yet empirically validated.

**What are the implications of the main findings?**

- SEED may help educators connect an observation with its significance and one prioritized action while inviting learner response; urgent safety action takes precedence.
- Both learner and educator perspectives, construct development, cultural adaptation, and comparative testing are required before routine implementation

**Abstract:**

Background: Clinical feedback occurs in hierarchical, time-pressured settings. Objective: This Perspective proposes relational wellness as a dyadic condition of a feedback episode and describes a brief feedback heuristic. Approach: The framework was developed before the focused, non-systematic searches used to check and align specific claims with sources. We compare SEED with established approaches and map its rationale, boundaries, and testable mechanisms. Key Propositions: In a relationally well feedback episode, learner and educator can remain respected and connected while engaging in truthful learning work. The author-developed SEED heuristic comprises See the situation and invite perspective; Explain observed performance and significance; Extend one prioritized next step collaboratively; and Dialogue to check understanding and invite response. Dignity governs all moves. Safety instructions may precede dialogue. SEED overlaps with Ask–Tell–Ask and remains unvalidated. Conclusions: Separate construct development should test the proposed level, dimensions, and discriminant validity of relational wellness using both participants’ perspectives. Co-design, technical and cultural review, feasibility work, and comparative evaluation are required before effectiveness claims.

## 1. Introduction

Clinical education is conducted through relationships as well as curricula. A medical trainee may defend a diagnostic plan during rounds before an unfamiliar team; a dental student may perform an irreversible procedure while a patient, assistant, and faculty member observe. In both settings, feedback must protect patients, identify performance gaps, and support learning within steep hierarchies and limited time. The interpersonal conditions surrounding that feedback influence whether uncertainty is discussed, whether errors are disclosed, and whether learners remain cognitively engaged [1,2,3,4,5]. In a longitudinal study of US medical students, mistreatment and perceptions of the learning environment were associated with subsequent burnout, empathy, and career regret [6]; that study concerns mistreatment and the broader learning environment rather than feedback specifically, and is cited as context only.

The effects of feedback are not uniformly beneficial. Foundational syntheses show that feedback is most useful when it clarifies the task, the current performance, and the next action, whereas feedback that redirects attention toward the self can impair subsequent performance [7,8]. Contemporary health professions scholarship has similarly moved away from a one-way delivery model toward educational alliance, learner agency, feedback literacy, coaching, and dialogue [9,10,11,12,13,14,15]. The relevant question is therefore not simply whether an educator gives feedback, but what the interaction permits the learner to understand, say, and do next.

We use relational wellness as a proposed lens on small interpersonal moments in clinical feedback. No neurochemical mechanism is inferred from a particular feedback phrase; claims about dopamine responses would require different evidence [16].

This Perspective has three objectives: (1) to propose a provisional definition of relational wellness and delineate its boundaries with neighboring constructs; (2) to position a proposed brief feedback heuristic against established approaches; and (3) to specify the heuristic’s theoretical warrants, boundaries, risks, and staged research agenda. The principal scope is brief formative feedback in undergraduate and postgraduate medical and dental clinical education. Patient counseling, peer support, mental-health intervention, and formal remediation are intentionally excluded as applications requiring separate evidence and validation.

## 2. Article Type and Approach to Concept Development

This Perspective presents a framework and heuristic developed by the authors before the literature searches reported here. Focused, non-systematic searches on 15–16 September 2026, undertaken after the framework and heuristic had been developed, were used to verify and align sources with pre-existing claims, identify countervailing evidence and comparators, and refine wording; they did not generate the framework. Three separate principal PubMed expressions were used: (“medical education” OR “dental education” OR “health professions education”) AND (feedback OR “feedback culture” OR “feedback literacy” OR coaching OR “educational alliance”); (“medical education” OR “dental education” OR “health professions education”) AND (“psychological safety” OR shame OR “relational well-being” OR “relational wellbeing” OR “learning environment”); and (“medical education” OR “dental education” OR “health professions education”) AND (culture OR “power distance” OR language OR linguistic OR bias OR equity). The principal ERIC expression was (“medical education” OR “dental education” OR “health professions education”) AND (feedback OR “feedback literacy” OR coaching OR “educational alliance” OR “psychological safety” OR culture OR bias OR equity). These are the principal expressions reported by the authors; database filters, result counts, and exact search logs were not retained and are therefore not reconstructed here.

This was a claim-driven source-selection process, with a risk of confirmation bias. Sources were retained when they informed a principal claim, construct boundary, comparator, risk, or hypothesized mechanism. Backward citation chaining examined references in selected foundational and relevant papers, and forward chaining sought later papers citing them; neither was logged as a comprehensive search. A final check on 28 September 2026 aligned citations with the revised wording, rather than serving as a fresh systematic search. The four authors discussed construct boundaries and revised the moves iteratively; the literature-identification method was led by R.K. No protocol, formal screening flow, or risk-of-bias appraisal was performed, and no learner, patient, or external expert participated in development. Every proposed mechanism and script remains an unvalidated hypothesis or illustration.

## 3. Relational Wellness: Definition, Level, and Boundaries

We propose relational wellness as a dyadic condition of a particular clinical feedback episode, in which the learner and educator can remain respected and connected while addressing observed performance truthfully and identifying a safe next action. The episode is the unit of analysis; each participant may perceive it differently. Repeated episodes, supervisory relationships, team climate, and institutional conditions may shape or result from this condition but are distinct levels. This provisional definition does not assume that either person feels comfortable or agrees with the assessment.

Psychological safety was first described at team level [1] and has been synthesized for healthcare teams [17], but it applies to more than team climate: it has been studied within workplace feedback conversations, including educators’ approaches that appeared to foster learner disclosure and productive engagement [2]. Our proposed focus is narrower in content, not necessarily in social scale: whether both parties can sustain respectful, connected, truthful work on a specific observed performance and next action. These features overlap with psychological safety and educational alliance [9]; a distinct construct has not been demonstrated. Belonging concerns acceptance and membership; relatedness in self-determination theory concerns connection as a basic need [18,19]; relationship-centered care considers relationships across care systems [20,21]. White’s account of relational well-being emphasizes social conditions rather than an individualized feeling [22]. Educators’ responses to ambiguity and the hidden curriculum may also shape whether trainees voice uncertainty [23].

The proposed distinction as positioned in Table 1, is a testable claim, not evidence of novelty: if episode-level relational wellness cannot be measured reliably or distinguished from episode-level psychological safety and educational alliance, it should be folded into those established constructs. Learner and educator reports, observations, and context measures will be needed to test this possibility.

## 4. What Feedback Evidence Supports—And Complicates

### 4.1. Task Focus, Specificity, and Possible Harm

Feedback is not inherently corrective. Kluger and DeNisi found that a substantial minority of feedback interventions reduced performance; their feedback-intervention theory proposes that performance suffers when attention shifts away from the task and toward the self [8]. Hattie and Timperley similarly organize effective feedback around three questions—where the learner is going, how the learner is going, and what comes next—and distinguish task, process, self-regulation, and self-level feedback [7]. These findings support observable, task-relevant language and a prioritized next action while challenging the assumption that warmer wording alone improves learning.

The proposed heuristic therefore avoids generic praise and identity labels. “You are naturally gifted” provides less usable information than “You identified the change in hemodynamics and revised the differential before prompting.” Specificity can direct attention toward behavior, reasoning, and standards. It cannot guarantee accuracy, fairness, or uptake.

### 4.2. Relationship, Dialogue, and Learner Agency

The educational-alliance literature reframes feedback as negotiation within a relationship rather than transmission from a credible expert to a compliant recipient [9]. Feedback literacy further emphasizes learners’ capacities to appreciate feedback, make judgments, manage affect, and take action, alongside educators’ and programs’ responsibilities to create usable feedback processes [10,11,12,13]. A study of medical students and residents also identifies feedback orientation as a learner attribute relevant to how feedback is taken up [24]. R2C2 and its in-the-moment adaptation operationalize relationship, reaction, content, and coaching through reflective dialogue and collaborative planning [14,15].

These perspectives expose a weakness in an educator-only script: observation, explanation, and direction are insufficient unless the learner can offer a perspective, test understanding, participate in planning, and revisit the change. SEED is therefore framed as an opening to dialogue. It is not a complete feedback cycle.

### 4.3. Emotion, Shame, and Accountability

Emotion influences attention and meaning, but the framework does not assume that discomfort is harmful or that emotional reassurance is always indicated. Shame becomes educationally relevant when attention shifts from a remediable behavior (“I omitted the allergy”) toward global identity threat (“I am unsafe and do not belong”). Shame experiences in medical training have been associated with concealment, withdrawal, and changes in professional identity [25]. A dignity-respecting response may reduce avoidable identity threat, but this remains a proposed mechanism rather than evidence of SEED effectiveness.

Accountability remains explicit. Safety-critical action must be stopped; standards and consequences must be named; repeated concerns require documentation and a formal process. Respecting dignity does not mean cushioning every message with praise, delaying an urgent instruction, or promising a favorable outcome.

## 5. Positioning SEED Among Established Approaches

Table 2 positions the proposed heuristic alongside established models. R2C2-in-the-moment has an empirical and implementation history [14,15]. Ask–Tell–Ask also opens with learner perspective, offers observer feedback, and returns to reflection and planning [26]. SEED proposes three incremental emphases for testing: explicitly connect the observation to clinical or educational significance; prioritize one immediately actionable next step; and apply dignity as a constraint throughout the exchange. These emphases may already occur in skilled Ask–Tell–Ask practice. Comparative study must test whether their explicit packaging adds value beyond familiar models or merely adds acronym burden.

SEED is also the acronym for the Supporting Educational Excellence in Diversity curriculum [29], which addresses a different educational purpose. We use “SEED dialogue heuristic” to identify this prototype; stakeholder co-design should consider a more distinctive name.

## 6. The Proposed SEED Dialogue Heuristic

SEED names four observable conversational functions as noted in Table 3, not four mandatory sentences or an invariant sequence. Dignity is a continuous constraint on S and both E functions as well as D. The distinctive D function is the educator’s explicit check of understanding and invitation to respond, correct the account, or negotiate follow-up after offering a next action. Dialogue can also occur earlier. In urgent hazards, a safety command takes precedence; learner perspective and the closing check can follow once the hazard is controlled.

### 6.1. A Brief Opening and the Required Continuation

For feasibility testing, the author-proposed opening target is approximately 30–90 s, not a validated threshold. A possible sequence is: “How did that go from your perspective? I observed [specific performance]. It matters because [standard, reasoning, or safety significance]. One next step could be [prioritized action]. What did you hear, and what would you try or change?” This resembles Ask–Tell–Ask [26]; SEED’s proposed explicit additions are the significance link, one prioritized action, and dignity as a constraint throughout. The template is not verbatim language and may be reordered for safety. Understanding, support, and follow-up must be checked.

### 6.2. Medicine and Dentistry: Shared Logic, Different Conditions

SEED is proposed for health professions feedback and illustrated in medicine and dentistry, without independent validation in either. Across settings, hierarchy, uncertainty, and patient-safety obligations can shape feedback. In the authors’ clinical illustrations as positioned in Table 4, chairside procedural teaching may involve direct patient observation and decisions that cannot simply be undone, and faculty continuity may vary across programs and rotations; these are contextual descriptions by the authors, not findings attributed to the grouped references. Dental education studies address learning climate [30], expectations about feedback [31], students’ use of feedback [32], and assessment concerns [33]; these sources do not establish every feature of a particular chairside exchange.

Therefore, examples must be technically reviewed within each discipline, and implementation should test whether the same move functions similarly across diagnostic, procedural, and longitudinal supervisory contexts.

## 7. Contextual Limits: Culture, Language, Equity, and Safety

### 7.1. Cultural and Linguistic Variation

Feedback is interpreted through professional, institutional, and cultural contexts. Learning cultures vary in their approaches to authority and correction [4,34]. The scoping review [35] draws on heterogeneous Asian settings, with variation both within studies and between settings; it does not describe a uniform “Asian” feedback culture or establish transfer to other geographic, linguistic, or cultural contexts. It discusses power distance and collectivism as relevant influences on feedback seeking and interpretation. The effects of specific English phrases, translation, and linguistic pragmatics on SEED remain hypotheses rather than established findings.

Cultural responsiveness therefore requires more than softening tone. Local learners and educators should co-design acceptable prompts and test how authority, disagreement, privacy, and directness influence interpretation in specific settings. Interpreters and bilingual experts should be involved when language adaptation is studied, and translated versions should undergo cognitive interviewing rather than literal substitution alone. Fidelity should be defined by function—observable evidence, significance, an actionable next step, and genuine opportunity for response—not by literal English wording. These recommendations are proposed adaptation principles requiring empirical testing; no claim of international or cross-linguistic generalizability is made.

### 7.2. Equity and Bias

Specificity may make feedback more auditable, but it does not eliminate bias. Narrative evaluations have shown racialized and gendered differences in descriptive language [36], and race-neutral educational structures can reproduce inequity [37]. Even a highly specific comment may reflect unequal observation opportunities, a biased standard, stereotype-based interpretation, or differential tolerance for error. Asset framing may broaden what evaluators notice, but it likewise requires attention to context and structural conditions [38].

Equitable use of SEED would require disaggregated audits of who receives observation, developmental challenge, coaching time, second chances, and formal escalation; calibration around evidence and standards; protected routes to contest inaccurate feedback; and examination of downstream decisions. The heuristic cannot correct structural inequity, mistreatment, inaccessible systems, or retaliation. Institutions remain responsible for those conditions.

### 7.3. When SEED Is Inappropriate or Insufficient

SEED should not delay commands during emergencies or an imminent procedural hazard. It is not a substitute for formal remediation, disciplinary or fitness-for-duty processes, documentation, due process, supervision, or mandatory reporting. It is also insufficient when a learner discloses hopelessness, impairment, harassment, discrimination, or danger; escalation routes vary by institution and jurisdiction and should follow local policy and emergency guidance.

Consent, shared decision making, teach-back, trauma-informed care, and interpreter-mediated communication are distinct clinical domains. Transfer of this educational feedback heuristic to patient care would require separate patient co-design and validation.

## 8. Staged Development and Research Agenda

Development of relational wellness as a construct and evaluation of SEED as a conversational heuristic as proposed in Table 5, are related but separate work streams. Neither should be treated as validated by the other. Broad implementation or claims about distal outcomes would be premature.

For the construct work stream, paired learner and educator ratings and independent observation should assess episode-level respect, connection, truthful participation, and agreement about next actions, alongside established comparator measures. Because SEED as written specifies educator behavior while relational wellness is defined as dyadic, both work streams should also record learner behaviors (for example, questions, corrections, and disagreement) and learner perceptions rather than inferring them from educator fidelity. For SEED, initial outcomes are specificity, significance comprehension, actionability, co-created plan quality, learner voice, educator experience, time, and fidelity. Record adverse outcomes including scripting, intrusion, obscured standards, suppressed disagreement, inequitable use, and delayed safety action. Distal outcomes should await construct and feasibility evidence.

## 9. Limitations

The framework was developed before the claim-driven searches. Their focused, non-systematic source selection risks confirmation bias, especially when judging the proposed construct against established ones. Search logs, filters, counts, comprehensive screening, and formal appraisal are unavailable. Medicine is better represented than dentistry among the cited feedback sources, and equivalence across disciplines is untested.

Relational wellness and SEED are author-proposed concepts. Neither has undergone learner or educator co-design, expert consensus, content validation, cognitive interviewing, cultural or linguistic adaptation, feasibility testing, reliability assessment, or outcome evaluation. The examples were written by the authors and may be experienced as formulaic, patronizing, intrusive, or technically inappropriate outside the stated context. The paper is written by US-based authors and may embed North American assumptions about hierarchy, individual recognition, and direct dialogue.

SEED overlaps substantially with established models and may not add enough value to justify another mnemonic. The acronym also collides with the Supporting Educational Excellence in Diversity curriculum [29]. The proposed sequence and mechanisms are hypotheses; all four moves may not be necessary, and a different name or structure may emerge through co-design. No causal, neurochemical, educational, well-being, or patient outcome can be inferred. Finally, respectful communication cannot compensate for workload, mistreatment, biased assessment, unsafe supervision, inadequate mental-health resources, or weak due-process and reporting systems.

## 10. Conclusions

Brief clinical feedback can be immediate and uncompromising while avoiding identity-level judgments. The proposed SEED functions invite perspective, explain evidence and significance, prioritize an action, and check understanding and response. Dignity applies throughout, and urgent safety instructions come first. Whether the proposed episode-level relational-wellness condition is distinct from established constructs remains an open empirical question.

This is an unvalidated prototype that substantially overlaps with existing approaches. Separate construct work and comparison with Ask–Tell–Ask and R2C2 should test whether the explicit significance link, prioritized action, and continuous dignity constraint produce useful differences without unintended effects.

## Figures and Tables

**Table 1:** Conceptual boundaries of relational wellness. The distinctions are author-developed and require empirical testing.

Construct	Proposed Level/Focus	Core Question	Relationship to Relational Wellness
Relational wellness (proposed)	Dyadic condition of one feedback episode, shaped by wider context	Can both participants remain respected and connected while addressing observed performance and a safe next action?	Provisional; requires dimensional and discriminant validation from both perspectives.
Psychological safety	Team, relationship, or feedback episode	Is interpersonal risk-taking safe here?	Overlaps at episode level [2]; a distinction based only on team versus dyad is untenable.
Educational alliance	Supervisor–learner relationship	Do both parties trust one another’s commitment to learning goals?	Provides the relational foundation for credibility and negotiated feedback.
Feedback literacy	Learner, educator, and program capacities	Can participants make sense of information and enact improvement?	Adds learner agency and the ability to use feedback beyond the moment of delivery.
Belonging/relatedness	Individual experience within a group or relationship	Am I accepted and connected here?	May be an antecedent or outcome, but is not guaranteed by a single respectful interaction.
Relationship-centered care	Patient–clinician, clinician–clinician, community, organization	How do relationships shape care and professional practice?	Broader intellectual lineage; the present paper is limited to educational feedback.

**Table 2:** Comparison of approaches. Evidence and limitation statements are the authors’ qualitative appraisal from the focused, non-systematic source-identification process, not formal evidence grades or comprehensive claims of uptake.

Approach	Purpose/Structure	Setting and Duration	Learner Agency	Evidence and Limitations	When SEED Would Not Add Value
R2C2/R2C2-in-the-moment [14,15]	Relationship; reaction; content; coaching; adapted prompts for brief encounters.	Assessment feedback and clinical coaching; short to extended.	Explicit reflection and co-created change plan.	Developed from theory and tested qualitatively across programs; requires conversational skill.	When R2C2 prompts and follow-up are already feasible.
Educational alliance [9]	Mutual goals, trust, and credibility within the supervisory relationship.	Longitudinal and episodic supervision.	Feedback is negotiated, not merely accepted.	Conceptual account of negotiated feedback [9]; not a stepwise script.	When the need is to repair or develop the supervisory relationship.
Feedback literacy [10,11,12,13]	Capacities to appreciate information, judge quality, manage affect, and act.	Across curricula and repeated cycles.	Central; learner must interpret and enact feedback.	Describes capacities across feedback cycles [10,11,12,13]; application varies by setting.	When a program needs a longitudinal feedback system rather than a brief opener.
Ask–Tell–Ask [26]	Learner self-assessment; specific observer feedback; learner reflection/questions and action planning.	Brief clinical teaching.	Explicit questions at beginning and end.	Described for targeted clinical feedback [26]; comparative effectiveness against SEED is unknown.	When a well-used Ask–Tell–Ask routine already achieves specific, dialogic feedback.
PEARLS/advocacy-inquiry [27,28]	Blend learner self-assessment, focused facilitation, directive teaching, and examination of frames.	Simulation debriefing; typically extended.	High; reasoning and frames are explored.	Established debriefing methods; facilitator training and time are needed.	During formal simulation debriefing or complex reflection.
SEED dialogue heuristic (proposed)	Invite perspective; explain evidence and significance; prioritize one next step; check response. Dignity applies throughout.	Author-proposed opening target of approximately 30–90 s for a time-pressured formative exchange; requires feasibility testing.	Invited when feasible; safety commands may precede or defer learner questions.	Author-developed and unvalidated; may feel formulaic; acronym collision exists.	Formal remediation, emergencies requiring commands, disciplinary action, or any setting already served by an established model.

**Table 3:** Evidence-to-heuristic map. All mechanisms and outcomes are hypotheses. Relational wellness is a proposed dyadic episode-level outcome, to be assessed separately from SEED fidelity through learner and educator perspectives and observation.

Move	Observable Educator Behavior	Theoretical Warrant	Hypothesized Proximal Outcome	Risks/Boundaries
S—See the situation and invite perspective	Attend to clinical urgency, setting, power, and emotion without claiming to know the learner’s internal state; ask a brief perspective question when feasible.	Educational alliance; psychological safety; feedback literacy [1,2,9,10,11,12,13].	More accurate shared understanding; learner voice becomes available.	May feel intrusive or delay urgent action; observation must not become mind-reading.
E—Explain observed performance and significance	Name behavior, reasoning, or result and connect it to a standard, patient risk, or learning goal.	Task/process feedback and attentional focus [7,8].	Clarity about the evidence and why it matters.	Specific language can still encode bias or be factually wrong; self-level labels remain harmful.
E—Extend one prioritized next step collaboratively	Offer or elicit one feasible next action, then check or negotiate it.	Feedback literacy; coaching; self-determination [10,11,12,13,14,15,18,19].	Reduced overload; actionable plan and appropriate autonomy.	One step may oversimplify complex gaps; educator must not conceal consequences.
D—Dialogue and check understanding (dignity throughout)	After a proposed action, ask what the learner heard, invite correction or disagreement, and agree on support or follow-up; respect inherent dignity throughout every move.	Educational alliance; shame literature; dialogic feedback [9,10,25].	Hypothesized continued truthful participation by both people; distinctness from psychological safety remains to be tested.	Can sound formulaic or patronizing; dignity-respecting language cannot substitute for fair systems.

**Table 4:** Author-developed, illustrative, and unvalidated examples for brief formative feedback. These are not clinical protocols.

Situation	Problematic Language	Possible SEED-Informed Opening	Boundary/Follow-Up	SEED Moves/Reason for Sequence
Medical: incomplete differential on rounds	“You should know this by now.”	“Your leading diagnosis fits the time course. A dangerous alternative is missing. Which diagnosis is it, and what finding would change today’s plan?”	Confirm understanding and revisit reasoning after rounds.	Reordered: brief E (what fits, what is missing) first; the question then elicits the learner’s reasoning and next step (S/E-extend); D deferred until after rounds because of the public, time-limited setting.
Medical: omitted allergy in handoff	“That was careless.”	“The allergy was omitted, creating a safety risk. Add it now. Where will you place the allergy check in your next handoff?”	Document/escalate according to severity and policy; do not substitute supportive wording for safety action.	E (observation, significance) and safety action first; the closing question begins the next-step plan (E-extend); S and D deferred until the hazard is controlled.
Dental: excessive crown-preparation taper	“This prep is unacceptable. Keep reducing until it looks right.”	“The axial convergence appears excessive. Stop further reduction. What was your intended path of insertion? Let us assess the remaining tooth structure, possible additional retentive features, and the appropriate restorative plan, with specialty input as needed.”	This is an illustration, not a treatment recommendation; specialty review is required before publication or teaching use.	E and safety stop first; a brief learner-perspective question follows immediately; plan (E-extend) and D deferred until remaining structure has been assessed.
Dental: local-anesthetic positioning	“You are doing it wrong again.”	“Before needle insertion, pause. Re-identify the landmarks and talk me through the target, angulation, depth, and safety checks.”	Use immediate unambiguous commands when safety requires; the teaching dialogue follows stabilization.	Safety command first (E abbreviated); “talk me through” elicits the learner’s reasoning once paused; full E/E and D after positioning is safe.
Either: learner becomes tearful	“Take a breath; you are too emotional.”	“I notice that you are tearful. Would you prefer a brief pause, or to tell me what you heard first? We can then discuss the concern and next step.”	Do not infer the cause, demand emotional suppression, or abandon standards.	S observation and choice first; E/E and D after pause if needed.
Either: repeated performance concern	“I keep telling you the same thing.”	“This concern has occurred in three documented encounters. Please tell me how you understand the pattern. We need a formal plan specifying the standard, practice, support, reassessment, and possible consequences.”	Use institutional remediation and due-process procedures; SEED is not the remediation process.	E (documented pattern) → S (invitation to give own account) → E (formal plan, not negotiated); D check not shown and expected at formal follow-up; formal process governs.

**Table 5:** Two linked, proposed research work streams: construct development for relational wellness and staged development for the SEED heuristic. Criteria are prospective examples to specify in protocols, not retrospective evidence of validity.

Stage	Purpose	Suggested Methods	Decision Criteria
A. Relational wellness construct development	Specify the operational definition, episode-level dimensions, observable indicators, and level of measurement (individual report, dyadic composite, or observed interaction); test whether a distinct construct is warranted.	Cognitive interviews and content mapping with learners and educators; paired episode reports plus observation; multilevel analysis of repeated episodes; convergent and discriminant tests against episode psychological safety, alliance, belonging, and well-being.	Pre-register dimensionality and level hypotheses, content-rating rubric and accept/revise rules; seek cross-perspective agreement where appropriate, reliability, and discriminant evidence; abandon the separate label if unsupported.
1. SEED: Co-design and concept refinement	Establish relevance, language, boundaries, and a distinctive name.	Learner and educator interviews/focus groups in medicine and dentistry; include varied identities, training levels, and cultural settings.	Clear construct boundaries; acceptable functional language; documented divergent views.
2. SEED: Expert and stakeholder content validation	Assess whether each move is necessary, clear, and technically accurate.	Assess each SEED move and example with learners, clinical educators, assessment and equity experts.	Before ratings, pre-specify the rating scale and item-level accept/revise/reject thresholds and missing-response rules; report ratings and revisions.
3. SEED: Cognitive interviewing and cultural adaptation	Identify interpretations, patronizing or intrusive language, and translation problems.	Think-aloud/cognitive interviews; bilingual review; purposive testing across settings with differing authority and communication norms.	Intended functions are understood without avoidable identity threat or ambiguity; translation and pragmatic effects are documented rather than assumed.
4. SEED: Usability, fidelity, and reliability	Determine whether users can identify and enact the moves.	Video vignettes, standardized encounters, behavioral coding, interrater reliability.	Acceptable fidelity, interrater agreement, time burden, and comprehension.
5. SEED: Feasibility and pilot evaluation	Assess acceptability and unintended effects in authentic settings.	Mixed-method pilots; conversation analysis; pre-specified balancing measures.	Feasible workflow; acceptable authenticity; no signal of obscured standards or delayed safety action.
6. SEED: Comparative evaluation	Determine whether SEED adds value over existing practice or another model.	Compare SEED with Ask–Tell–Ask, R2C2-in-the-moment, or usual feedback in randomized simulation or pragmatic designs; isolate significance explanation, single-step prioritization, and dignity constraint in process coding.	Pre-specified proximal outcomes and adverse effects; transparent null or negative findings.
7. SEED: Program-level study	Examine sustained use and equity only after earlier stages.	Longitudinal multi-site studies with disaggregated process and climate measures.	Evidence of benefit, implementation fidelity, cultural fit, and equitable distribution.

## Data Availability

No new data were created or analyzed in this study. Data sharing is not applicable to this article.
